# Validation of the Index for Facial Angiofibromas: Data analysis from a randomized controlled trial of sirolimus gel treatment in patients with tuberous sclerosis complex

**DOI:** 10.1111/1346-8138.17220

**Published:** 2024-04-15

**Authors:** Izumi Hamada, Yoshinori Yukutake, Yusuke Morita, Norifumi Ishikawa, Kenji Shimizu, Mari Wataya‐Kaneda

**Affiliations:** ^1^ Research & Development Division, Nobelpharma Co., Ltd Tokyo Japan; ^2^ Department of Neurocutaneous Medicine, Division of Health Sciences Graduate School of Medicine Osaka University Osaka Japan; ^3^ Department of Dermatology, Graduate School of Medicine Osaka University Osaka Japan

**Keywords:** angiofibroma, sirolimus, topical administration, tuberous sclerosis, validation study

## Abstract

The Index for Facial Angiofibromas (IFA), a novel scoring system for angiofibromas, has been validated in patients with tuberous sclerosis complex (TSC). The objective of this analysis was to further validate the IFA using data from a clinical trial of topical sirolimus in patients with TSC. This was an analysis of photographs from a Phase III trial conducted in Japan (NCT02635789). Patients (*n* = 62) were randomized 1:1 to receive sirolimus or placebo gel for 12 weeks. Changes in angiofibromas were independently assessed using the primary composite endpoint, the Facial Angiofibroma Severity Index (FASI), and the IFA. Thresholds for a clinically meaningful change in IFA score were evaluated using receiver operating characteristic (ROC) analysis. The IFA scores had good‐to‐excellent inter‐assessor reliability, very high intra‐assessor reliability, and could be used to evaluate the distribution of disease severity at baseline. High correlations were observed between the categorized change from baseline in IFA scores and the primary composite endpoint (Kendall's coefficient of concordance, *W* = 0.8655, *p* < 0.0001), and between the change from baseline in IFA and FASI scores (Kendall's coefficient of concordance, *W* = 0.745, *p* < 0.0001). By ROC analysis, an optimal IFA cut‐off point of 1.667 was determined to distinguish patients with markedly improved or improved angiofibromas from those with slightly improved or unchanged angiofibromas (area under the curve 0.937) as determined by the primary composite endpoint. The IFA score is potentially clinically useful because of its high validity and reliability. A decrease in score from baseline of ≥1.667 may be considered clinically meaningful.

## 1 INTRODUCTION

Tuberous sclerosis complex (TSC) is a rare, autosomal dominant, inherited disorder characterized by hamartomas throughout the body.^1^ It is caused by mutations in the *TSC1* and *TSC2* genes, resulting in dysfunction of the tuberin–hamartin complex. This leads to constant activation of the downstream mechanistic target of rapamycin (mTOR complex 1), promoting cell growth and proliferation, and inhibiting apoptosis.^2^ Facial angiofibromas are one of the main skin manifestations of TSC, affecting approximately 75% of patients. These hamartomas result from an increase in the connective tissue and vascular components of the skin.^3^

Topical sirolimus, which is an mTOR complex 1 inhibitor, has been shown to be safe and effective for the treatment of facial angiofibromas.^4^, ^5^, ^6^ A gel formulation was approved for use as a topical treatment for TSC skin lesions in Japan in 2018,^5^ by the United States Food and Drug Administration in March 2022,^7^ by the National Medical Products Administration in China in March 2023,^8^ and by the European Medicines Agency in May 2023.^9^ The approval of sirolimus gel was based on the results of a Phase III, randomized controlled trial that demonstrated significant composite improvements in the size and color of angiofibromas with sirolimus gel compared with placebo after 12 weeks of treatment.^6^ The primary endpoint of this trial was based on the relative changes between pre‐ and post‐treatment angiofibroma severity for each patient. However, this endpoint, focusing on relative changes from the baseline, had a limitation in that the absolute severity of the angiofibroma at baseline and the magnitude of improvement could not necessarily be determined.

Several scoring systems have been developed to provide a quantitative evaluation of facial angiofibromas. The Facial Angiofibroma Severity Index (FASI) has been developed and validated for the assessment of facial angiofibroma lesions;^10^ however, for this system, smaller changes are difficult to detect and score changes may not be observed for mildly affected patients.^11^ Subsequently, the Index for Facial Angiofibromas (IFA) was designed and validated^11^ to address the limitations of the FASI. The IFA is an eight‐item score that focuses on the angiogenic component of angiofibromas as well as the extent of the lesions and the facial areas affected. However, these results need to be confirmed using a larger sample size, and the utility of the IFA needs to be clarified in relation to the traditional scoring systems described above. The objective of this analysis was to confirm that the IFA score is a meaningful scoring method by comparing and examining the correlations and characteristics between the IFA score and other evaluation methods. In addition, thresholds for clinically meaningful changes in IFA scores were determined.

## 2 METHODS

In this study, the photographs taken during a previously reported Phase III trial (NCT02635789)^6^ were evaluated using the IFA and FASI. The results were validated using statistical methods along with the results of the Phase III trial primary endpoint.

### 2.1 Outcome measures

#### 2.1.1 Independent Review Committee‐reviewed composite endpoint

The protocol‐defined primary endpoint for the previously reported Phase III trial was a composite endpoint of improvement in angiofibromas assessed by an Independent Review Committee (IRC) comprising three dermatologists.^6^ Photographs taken at baseline and 12 weeks after the start of treatment were examined and improvement was determined using a 6‐point scale ranging from “markedly improved” to “exacerbated” compared with baseline (scoring criteria for the primary endpoint are shown in Table S1).

#### 2.1.2 IFA

The change in IFA score from baseline to week 12 was determined by a re‐evaluation of the existing baseline and week 12 original photographs for each patient. This assessment was performed by an Independent Evaluation Committee (IEC), which was separate from the IRC that evaluated the photographs for the primary endpoint. The IEC was composed of three trained clinical experts in the field of dermatology. Before reviewing the photographs, all IEC assessors underwent a live training session on the assessment and scoring of angiofibroma lesions. IEC assessors could start patient evaluation only when they had successfully completed the training session.

The IFA is an eight‐item score that evaluates erythema (maximum score of 2), redness of angiofibroma (maximum score of 2), extent of red angiofibroma of all affected areas (maximum score of 3), diameter of largest angiofibroma (maximum score of 3), alar facial groove affected (maximum score of 1), percentage of nose affected (maximum score of 3), percentage of cheeks affected (maximum score of 3), and percentage of chin affected (maximum score of 3). Individual items and scoring criteria are listed in Table S2. The IFA total score (0–20) is the sum of all item scores with higher scores denoting more pronounced angiofibroma lesions. All three IEC assessors reviewed three photographs (front, left, and right side of the face) for each patient and visit. All assessors were blinded to patient identification, treatment assignment, and composite endpoint result. Assessors saw patient photographs taken at baseline and week 12 together on the screen, but these were presented in a random order as Visit A and Visit B to preserve blinding. Duplicate photos for seven randomly selected patients (approximately 10% of all photos) were included to assess intra‐assessor variability. The mean of the IFA scores across the three IEC members per patient and visit was used for data analysis.

#### 2.1.3 FASI

The FASI is a three‐item score that evaluates erythema and the size and extent of angiofibroma lesions.^10^ Of a maximum possible score of 9, scores of ≤5, 6–7, and ≥8 denote mild, moderate, and severe angiofibroma, respectively. As the FASI scoring system had only been validated outside Japan when this Phase III trial was conducted, one of the original IRC members also re‐assessed the original photographs collected for each patient using the FASI. Three photographs (front, left, and right side of the face) were assessed for each patient and visit. The assessor was blinded to patient identity, treatment assignment, and the composite endpoint result.

### 2.2 Statistical methods

All analyses were conducted using the full analysis set (FAS), which was specified in the Phase III trial as the group of patients who received the study drug and underwent a post‐baseline efficacy assessment. IFA total score at baseline and week 12, and the change from baseline to week 12 were summarized. The change from baseline to week 12 for the IFA total score was analyzed using the Wilcoxon rank sum test.

The change from baseline to week 12 in IFA total score was summarized according to the categories used for the primary analysis. Kendall's rank correlation coefficient and the coefficient of concordance and their associated *p*‐values were used to assess the correlation between the IFA score and the categories used for the primary analysis and the correlation between the IFA and FASI scores.

Intra‐assessor reliability was evaluated by the inclusion of additional duplicate photographs during scoring, and weighted kappa coefficients were calculated for each assessor. For the evaluation of inter‐assessor reliability, the correlation between the three IEC assessors in IFA total scores at baseline and week 12 was assessed using the intraclass correlation coefficient (ICC), according to the method established by Shrout and Fleiss.^12^

Receiver operating characteristic (ROC) curve analyses were performed to establish clinically significant changes in the IFA score that could be used to distinguish among the following outcomes in the primary endpoint of composite improvement in angiofibromas: (1) markedly improved or improved versus slightly improved or unchanged; (2) markedly improved versus improved, slightly improved, or unchanged; and (3) markedly improved, improved, or slightly improved versus unchanged.

## 3 RESULTS

### 3.1 Patients

This analysis was performed on the FAS from the primary analysis. All 62 enrolled patients (sirolimus gel, *n* = 30; placebo, *n* = 32) were included in the FAS. All patients received investigational drugs for 12 weeks and completed a 4‐week follow‐up and no patient discontinued treatment. Patient baseline characteristics have been reported previously. Patients were a mean age of 22.5 years (range, 6–53 years) and treatment groups were balanced.^6^

### 3.2 Correlation between IFA total score and the composite endpoint

**FIGURE 1:**
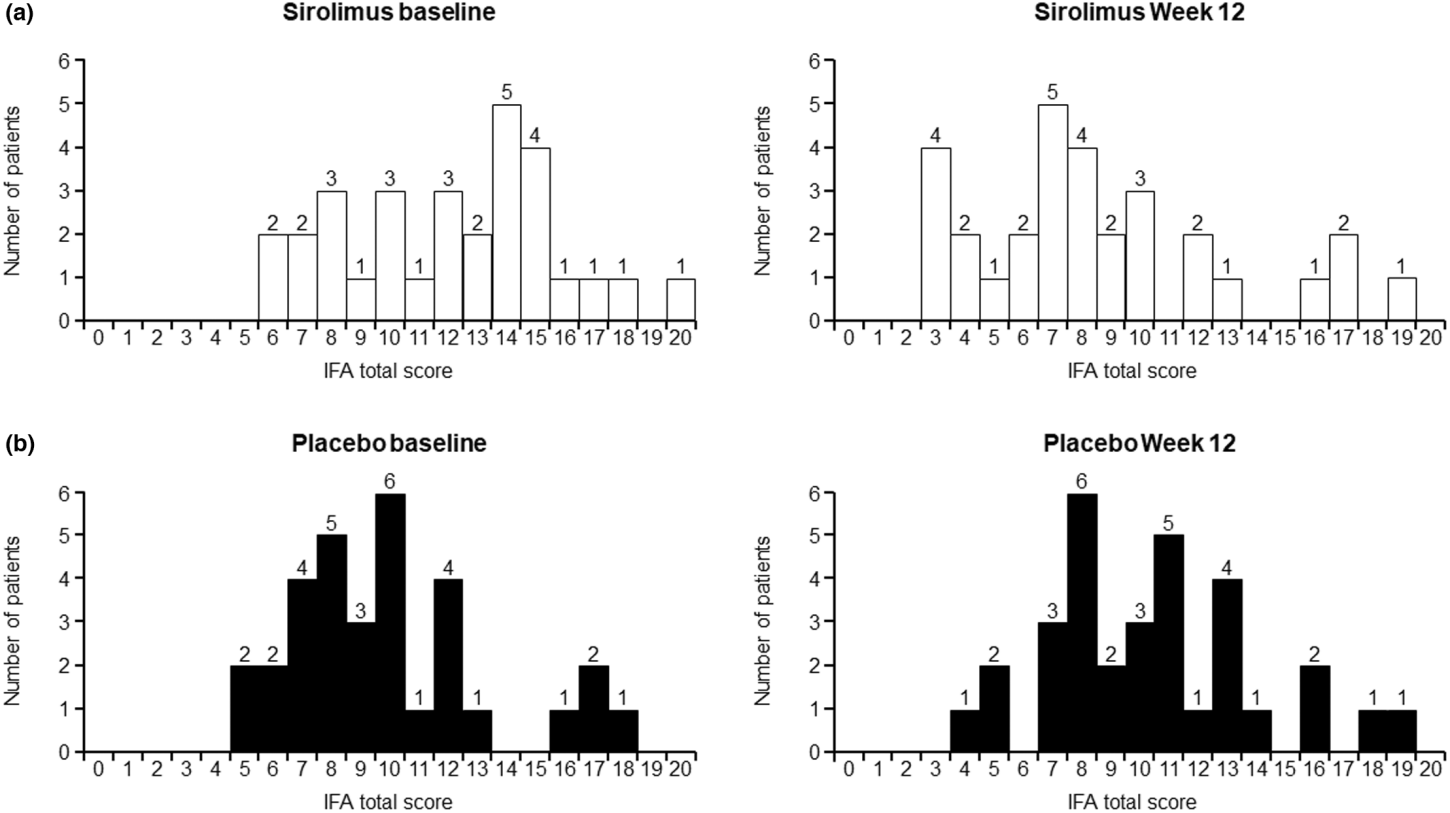
Distribution of Index for Facial Angiofibromas (IFA) scores by visit and treatment with (a) sirolimus and (b) placebo. For each patient, the IFA score was calculated as the mean of the scores reported by the three Independent Evaluation Committee members.

The mean ± standard deviation (SD) IFA total score at baseline was 12.1 ± 3.69 in the sirolimus group and 9.9 ± 3.43 in the placebo group (*p* < 0.001). Distribution scores at baseline and at week 12 are shown in Figure 1.

**FIGURE 2:**
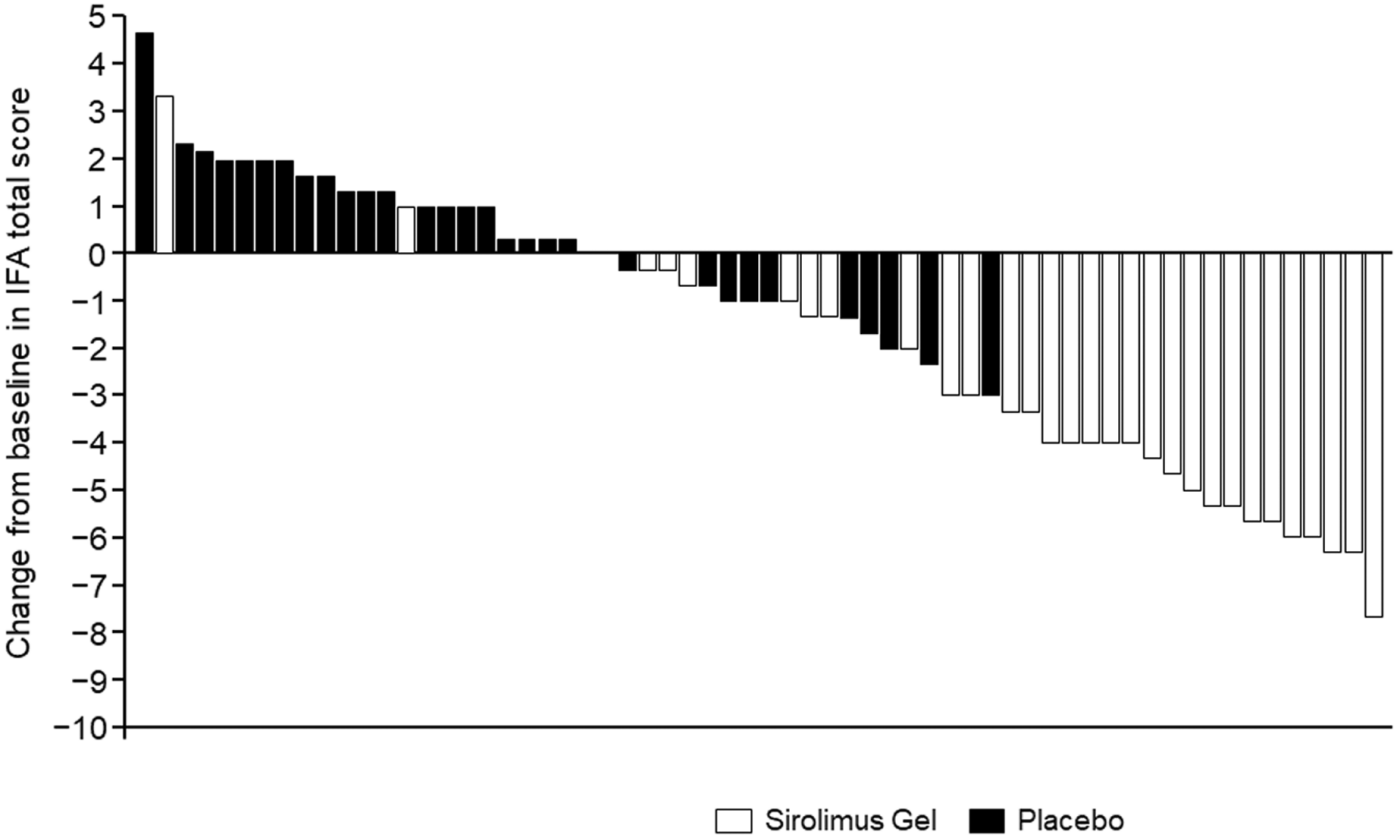
Waterfall plot for change in Index for Facial Angiofibromas (IFA) score from baseline to week 12 for all treated patients. For each patient, the IFA score was calculated as the mean of the scores reported by the three Independent Evaluation Committee members.

At week 12, the IFA total score had decreased by 3.5 points (SD 2.50) to 8.6 (SD 4.32) for sirolimus‐treated patients and increased by 0.5 points (SD 1.63) to 10.4 (SD 3.62) for placebo‐treated patients (Wilcoxon rank sum test, *p* < 0.001). The change from baseline to week 12 in IFA total score is shown for each patient in a waterfall plot (Figure 2).

**FIGURE 3:**
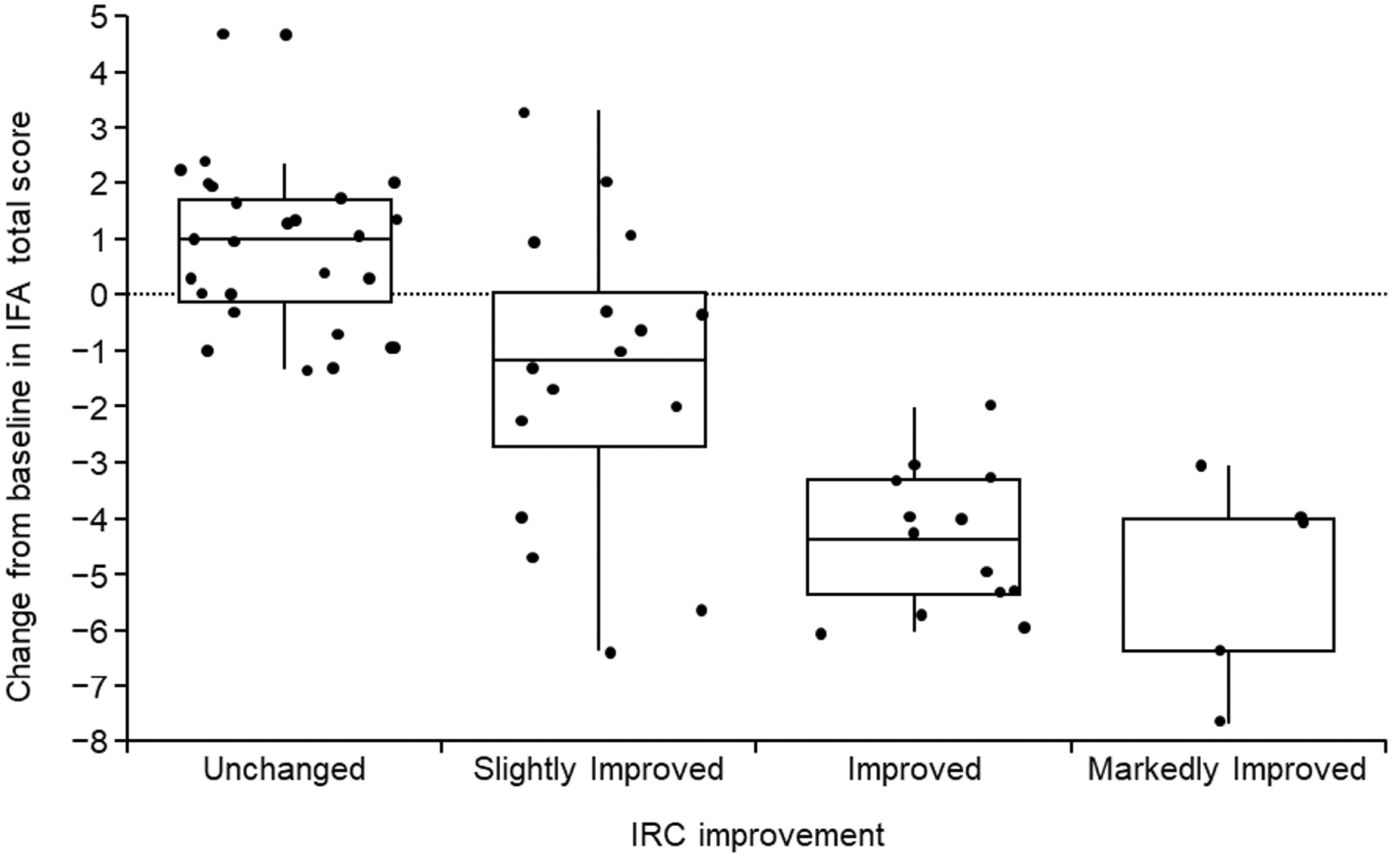
Box plots of improvement in Index for Facial Angiofibromas (IFA) score in categories defined by composite improvement of angiofibroma as determined by the Independent Review Committee (IRC). Box plots show the median, interquartile range and minimum and maximum values. For each patient, the IFA score was calculated as the mean of the scores reported by the three Independent Evaluation Committee members.

The changes in mean IFA total scores were calculated in categories defined by composite improvement of angiofibroma at week 12 as determined by the IRC. For patients with unchanged angiofibromas, the IFA total score worsened by a mean of 0.7. Conversely, mean improvements in IFA total scores of 1.4, 4.4, and 5.0 were observed for patients with slightly improved, improved, and markedly improved angiofibromas, respectively (Figure 3).

High correlations were identified for the categorized change from baseline in IFA score and the composite endpoint (Kendall's coefficient of concordance, *W* = 0.8655, *p* < 0.0001).

### 3.3 Correlation between IFA total score and the FASI score

**FIGURE 4:**
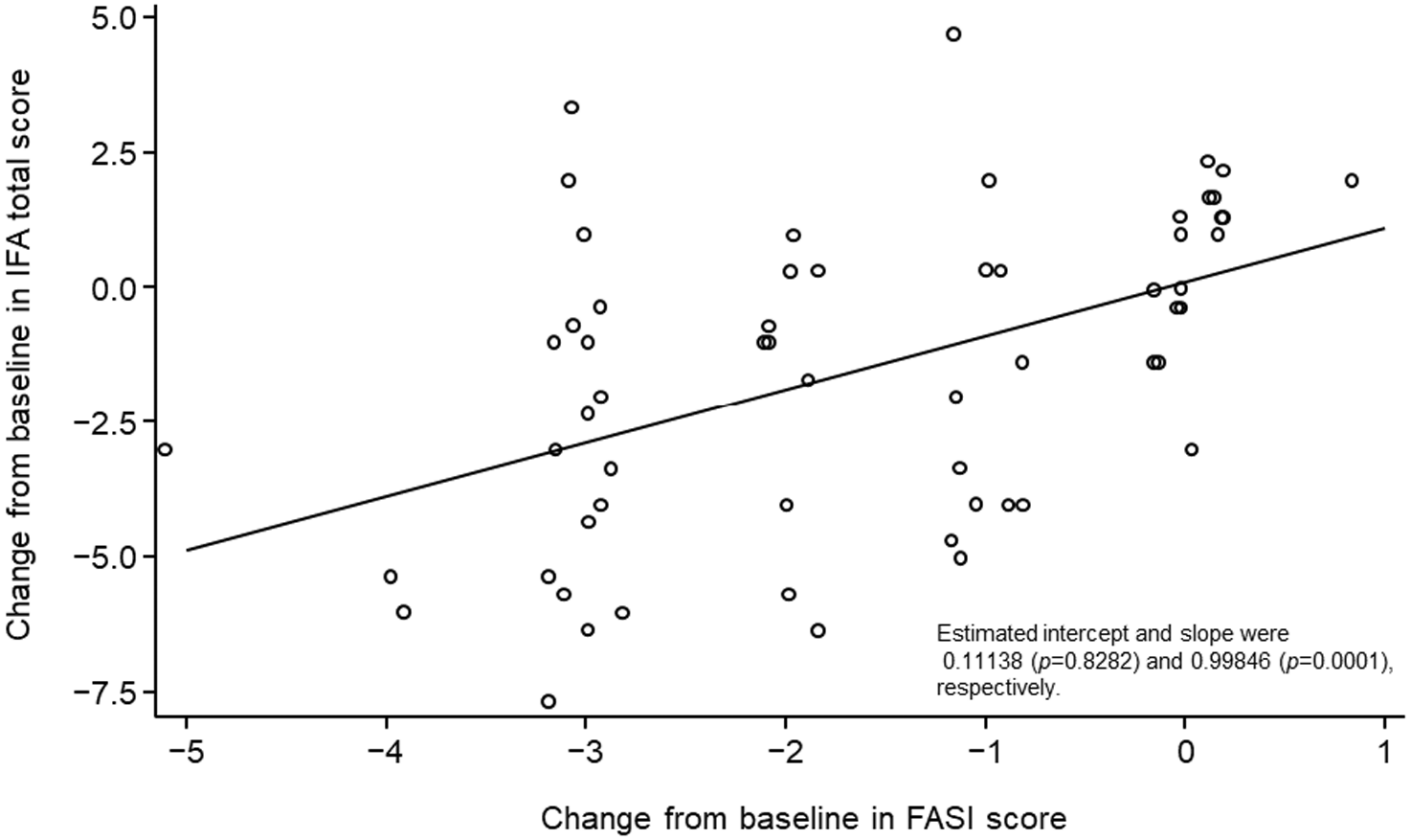
Scatter plot showing change from baseline to week 12 in Index for Facial Angiofibromas (IFA) score versus change from baseline to week 12 in Facial Angiofibroma Severity Index (FASI) score. For each patient, the IFA score was calculated as the mean of the scores reported by the three Independent Evaluation Committee members.

High correlations were also identified for the change from baseline in IFA total score and the change from baseline in FASI score (Kendall's coefficient of concordance, *W* = 0.745, *p* < 0.0001) (Table 1 and Figure 4).

**TABLE 1:** Correlation of change from baseline at week 12 in FASI score and IFA total score.

	Change from baseline			Kendall's coefficient of concordance	Kendall's rank correlation coefficient
	*n*	Mean	SD		
FASI score	60^a^	1.633	1.378	*W* = 0.745	*T* = 0.382
IFA score	62	1.422	2.873	*p* < 0.0001	*p* < 0.0001

Abbreviations: FASI, Facial Angiofibroma Severity Index; IFA, Index for Facial Angiofibromas; SD, standard deviation.

^a^ Two cases were excluded because they were not considered evaluable due to halation or difficulty in assessing lesions.

### 3.4 Assessment of intra‐ and inter‐assessor reliability

The weighted kappa coefficients for the IFA total score for the three IEC assessors were 0.85, 0.58, and 0.53, respectively, indicating very high intra‐assessor consistency for one assessor and moderate consistency for the other two. The correlation of IFA total scores among the three IEC assessors was high at both baseline and week 12. The observed ICC at baseline (0.77) and week 12 (0.83) can be considered excellent according to guidelines developed by Cicchetti,^13^ which state that an ICC of 0.75–1.00 reflects excellent correlation, or good according to guidelines developed by Koo and Li,^14^ which state that 0.75–0.90 reflects good correlation.

### 3.5 Clinically significant changes in IFA

**FIGURE 5:**
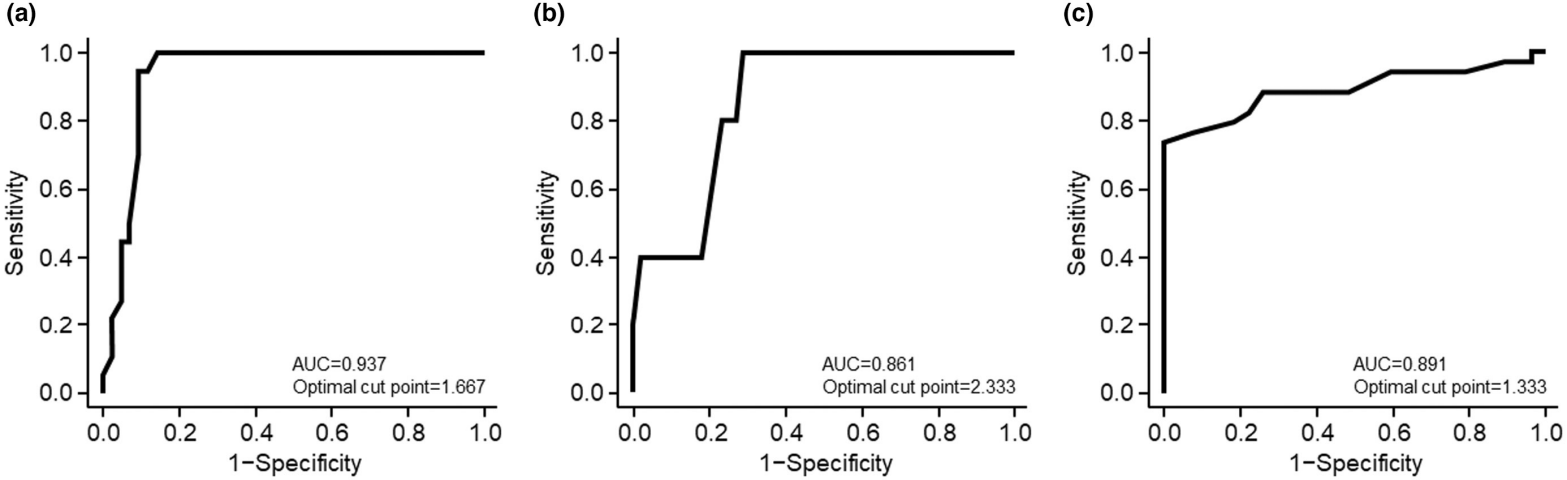
Change from baseline in Index for Facial Angiofibromas (IFA) score and composite improvement of angiofibroma (receiver operating characteristic analyses). Three analyses were performed: (a) markedly improved or improved versus slightly improved or unchanged, (b) markedly improved versus slightly improved or unchanged, and (c) markedly improved, improved, or slightly improved versus unchanged. AUC, area under the receiver operating characteristic curve.

Three ROC analyses were performed to identify optimal cut‐off points for the change in IFA total score from baseline to establish clinically significant changes (Figure 5). To distinguish patients with markedly improved or improved angiofibromas from those with slightly improved or unchanged angiofibromas, the optimal IFA cut‐off point was 1.667, resulting in an area under the curve (AUC) of 0.937 (Figure 5a). To distinguish patients with markedly improved angiofibromas from those with improved, slightly improved, or unchanged angiofibromas, the optimal IFA cut‐off POINT was 2.333, resulting in an AUC of 0.861 (Figure 5b). To distinguish patients with markedly improved, improved, or slightly improved angiofibromas from those with unchanged angiofibromas, the optimal IFA cut‐off point was 1.333, resulting in an AUC of 0.891 (Figure 5c).

## 4 DISCUSSION

In this analysis, the IFA scores showed good‐to‐excellent inter‐assessor reliability and very high intra‐assessor reliability. In addition, the IFA allowed for the assessment of baseline severity and the scores strongly correlated with traditional evaluation methods (i.e., composite improvement in angiofibroma and FASI), and clinically significant thresholds for the IFA score were established by ROC analysis.

Using data from a Phase III, randomized controlled trial, high correlations were observed between the categorized change from baseline in IFA scores and the composite primary endpoint (Kendall's coefficient of concordance, *W* = 0.8655, *p* < 0.0001) as well as between the change from baseline in IFA scores and the change from baseline in FASI scores (Kendall's coefficient of concordance, *W* = 0.745, *p* < 0.0001). The results of this analysis suggest some clinical validity in the use of the IFA for evaluating fibrous angiofibromas with a decrease in IFA score from a baseline of ≥1.667 successfully distinguishing patients with a composite endpoint of improved or better from those with slightly improved or unchanged angiofibromas. For example, an improvement of ≥2 points in the composite endpoint means that there is an improvement of 50%–75% of the lesion extent from baseline and that redness is fading. This would correspond to a general shrinkage or flattening of the lesion (approximately 50%–75% of the lesion size at baseline) and fading redness or partial disappearance of the lesion and significant fading of the redness. Previous research has indicated that a visible improvement would correspond to a 2‐point reduction in the IFA score,^11^ based on the intra‐assessor variance of 0.863 obtained through the Shrout and Fleiss^12^ ICC (2,1) model, in which the same assessor should recognize a true improvement of 2 points in 87% of cases. Although the cut‐off point obtained in this study is lower at 1.667, its potential validity is supported by the fact that it was obtained through comparison with traditional indices.

The results of this analysis also suggest that the IFA score may be a useful assessment method in addition to the composite endpoint as it is able to assess disease severity at baseline. This is particularly relevant given the observed differences in baseline disease severity between the sirolimus and placebo groups in the Phase III trial, despite randomization. Such differences underscore the importance of considering baseline severity to achieve a more accurate assessment of treatment efficacy. This might be attributed to the limited number of established evaluation tools for facial angiofibromas at baseline. The IFA may be a more sensitive scoring system for detecting smaller and earlier improvements in angiofibromas.

## 5 LIMITATIONS

This study had some limitations. First, TSC is a rare disease and, as such, the study population was small. Second, the study was limited to a Japanese population and may not be generalizable to a broader population. Finally, no case of exacerbation was identified and, therefore, changes in IFA scores in the case of exacerbation could not be assessed. To address these limitations, it would be beneficial to conduct a trial in a larger and more diverse patient population.

## 6 CONCLUSIONS

Our results support a previous validation study^11^ and showed that the IFA scoring system had good‐to‐excellent inter‐assessor reliability and very high intra‐assessor reliability. The IFA is useful in assessing the response of patients to treatment in a clinical setting as it accurately measures the clinical severity of facial angiofibromas associated with TSC and can detect small changes in symptoms.

## CONFLICT OF INTEREST STATEMENT

Izumi Hamada, Yoshinori Yukutake, Yusuke Morita, Norifumi Ishikawa, and Kenji Shimizu are employees of Nobelpharma Co., Ltd. Mari Wataya‐Kaneda is the holder of an endowed professorship funded by Nobelpharma Co., Ltd.

## Supporting information


Tables S1–S2.
