# Supplementary material for: Validation of the Index for Facial Angiofibromas: Data analysis from a randomized controlled trial of sirolimus gel treatment in patients with tuberous sclerosis complex
Source: J Dermatol. 2024 Apr 15;51(6):752–8. doi: 10.1111/1346-8138.17220 (PMC11483935; doi:10.1111/1346-8138.17220)
Supplement: Supplementary file 1 — Tables S1–S2. [file JDE-51--s001.docx]

**Supporting information**

**Validation of the Index for Facial** **Angiofibromas: Data analysis from a randomized controlled trial of sirolimus gel treatment in patients with tuberous sclerosis complex**

**Table S1.** Protocol-defined scoring criteria for primary endpoint of change in angiofibroma lesions from baseline

| Score | Degree of improvement | Criteria |
| --- | --- | --- |
| 3 | Markedly improved | Shrinkage, flattening, or disappearance of tumors is observed overall. A large decrease in the intensity of redness or a change in redness to the level equal to that of the normal region is observed nearly overall. |
| 2 | Improved | Shrinkage or flattening of tumors and a decrease in the intensity of redness are observed nearly overall, or disappearance of tumors and a large decrease in the intensity of redness is partially observed. |
| 1 | Slightly improved | Shrinkage or flattening of tumors and a decrease in the intensity of redness are partially observed, or a slight decrease in the intensity of redness is observed nearly overall. |
| 0 | Unchanged | There is no definite change in the size or the redness of tumors |
| –1 | Slightly exacerbated | Enlargement or new formation of tumors and an increase in the intensity of redness are partially observed, or a slight increase in the intensity of  redness is observed nearly overall. |
| –2 | Exacerbated | An enlargement or new formation of tumors is observed nearly overall, or a huge enlargement of tumors and an increase in the intensity of redness are partially observed, or more severe exacerbation is observed. |

**Definition of terms**

Overall: Not less than about 75% of the extent of the lesion at baseline; Nearly overall: About 50% to 75% of the extent of the lesion at baseline; Partially: About 25% to 50% of the extent of the lesion at baseline; Large decrease in intensity of redness: Change of ≥3 levels in redness in accordance with the Pantone^®^ color sample; Decrease/increase in intensity of redness: Change of ≥2 levels redness in accordance with the Pantone^®^ color sample; Slight decrease/increase in intensity of redness: Changes of 1 level in redness in accordance with the Pantone^®^ color sample.

**Table S2.** IFA scoring system – individual items of assessment and their scoring

| Item | Score 0 | Score 1 | Score 2 | Score 3 | Maximum score |
| --- | --- | --- | --- | --- | --- |
| Erythema | None | Light red | Marked red | - | 2 |
| Redness of AF | None | Light red | Marked red | - | 2 |
| Extent of red AF of all affected areas | None | Sporadic | <50% | >50% | 3 |
| Diameter of largest AF | None | <3 mm | 3–10 mm | >10 mm | 3 |
| Alar facial groove affected | No | Yes | - | - | 1 |
| Nose | None | <50% | >50% | Cluster | 3 |
| Cheeks | None | <50% | >50% | Cluster | 3 |
| Chin | None | <50% | >50% | Cluster | 3 |

AF, angiofibroma; IFA, Index for Facial Angiofibromas.
